# The complete mitochondrial genome sequence of *Sinomicrurus peinani* (Serpentes: Elapidae)

**DOI:** 10.1080/23802359.2022.2080011

**Published:** 2022-06-10

**Authors:** Sheng-bo Zhou, Zhao-bo Zhang, Zhi-he Zhang, Xin-yu Liu, Ping Guan, Bo Qu

**Affiliations:** a College of Bioscience and Biotechnology, Shenyang Agricultural University, Shenyang, PR China; b Key Laboratory of Global Changes and Biological Invasions, Shenyang, PR China; c College of Animal Science and Veterinary Medicine, Shenyang Agricultural University, Shenyang, PR China; d School of Life Science, Liaoning Normal University, Dalian, PR China; e Foreign Language School, Wuhan University of Technology, Wuhan, PR China

**Keywords:** *Sinomicrurus peinani*, mitochondrial genome, phylogenetic tree, Elapidae

## Abstract

*Sinomicrurus peinani* is a new species of the genus *Sinomicrurus* (Serpentes: Elapidae) from China and Vietnam in 2020. In this study, we successfully sequenced mitochondrial genome of an individual *S. peinani.* The complete mitochondrial genome of *S. peinani* is a circular molecule with the entire length of 19,477 bp. The base composition is T (28.1%), G (11.9%), and GC (38.5%), which contains two ribosomal RNA (rRNA) genes, 22 transfer RNA (tRNA) genes, 13 protein-coding genes, one origin of replication gene (D-loop), and two non-coding control regions, an origin of light-strand replication, and a 2346 bp non-coding region between tRNA-N and tRNA-Y. A maximum-likelihood (ML) tree of *S. peinani* and 13 other related species was constructed. The DNA data presented here will be useful to study the evolutionary relationships and genetic diversity of *S. peinani*.

*Sinomicrurus* Slowinski, Boundy, and Lawson, 2001 is a group of small proteroglyphous venomous snakes of the family Elapidae which are widely distributed in eastern and southeastern Asia (Slowinski et al. 2001; Zhao 2006). As they are small and secretive, there is little basic biological information (Wang et al. 2015). Only one complete mitochondrial genome was reported on this genus . *S. peinani* is a new species that was described by morphological and molecular differences in Guangxi, China and Vietnam in 2020 (Liu et al. 2020). In this study, we characterized the complete mitochondrial genome of *S. peinani* in order to obtain the basic mitochondrial information of this species.

A specimen of *S. peinani* was collected on 27 March 2021 from Cangwu County, Wuzhou City, Guangxi Province, China (N23.65°, E111.57°). It is 534.59 mm in length and is an adult female. Muscle tissue was extracted and immediately stored in 95% ethanol solution. DNA was extracted and paired-end sequencing was performed with Illumina Hiseq sequencing technology to construct the Illumina PE library. As there were some low-quality data in Illumina's original sequencing data, in order to make the subsequent assembly more accurate, we cut the quality of the original data: The adapter sequence in reads was removed; the base containing non AGCT at the 5′ end was removed before shearing; the ends of reads with low sequencing quality were trimmed (the sequencing quality value was less than Q20); reads containing 10% N was removed; the small fragments with the length less than 50 bp were discarded after the trimming of adapter and quality.

The complete mitochondrial genome was obtained by bioinformatics analysis after the quality control was conducted on the obtained sequencing data (Albertsen et al. 2013). The specimen and tissue sample are now stored in Bioscience and Technology College, Shenyang Agricultural University (specimen number: SYAUA21015).

The complete mitochondrial genome is a circular double stranded DNA, 19,477 bp in length. It consists of two ribosomal RNA (rRNA) genes, 22 transfer RNA (tRNA) genes, 13 protein-coding genes, two nearly duplicate H-strand (D-loop) genes, as most other advanced snakes, an origin of L-strand (OL) between tRNA-N and tRNA-C, and a 2346 bp non-coding region between tRNA-C and tRNA-Y. The total base composition of *S. peinani* genome is T (28.1%), G (11.9%), and the overall GC content is 38.5%. The complete mitochondrial genome reflects the typical characteristics of advanced snakes (Kumazawa et al. 1998), with 29 mitochondrial genes encoded on the H-strand except for the ND6 gene and eight tRNA genes which are encoded on the L-strand. The two rRNA contains rRNL (1479 bp) and rRNS (926 bp) separated by tRNV-TAC (66 bp), locating between ND1 (955 bp) and tRNF-GAA (62 bp). The 22 tRNA genes can carry 26 different kinds of amino acids and each RNA is 58–73 bp long. The 13 protein-coding genes contain seven kinds of NADH dehydrogenase subunit (ND1–ND6) and a NADH dehydrogenase subunit 4L (ND4L), three cytochrome c oxidase subunit (COX1–COX3), two ATP synthase F0 subunit (ATP6 and ATP8) and cytochrome b (CYTB). The two non-coding control regions are 994 bp and 1026 bp, respectively, and are 99.6% identical (other than length, they differ only by 4 bp). The 2346 bp non-coding region between tRNA-C and tRNA-Y is only known in one other advanced snake, *Sibon nebulatus*, which has a similar non-coding region in the same place of 5702 bp in length (GenBank EU728583; Mulcahy and Macey 2009). The mt-genome presented here also contains an origin of light-strand replication (OL: TTTCTCCGTTTTTGGAGGGGGGGAAAAAAACGGAGAAA), as most other squamates, between tRNA-N and tRNA-C.

In order to verify the newly determined mitochondrial gene sequence, we selected 13 related species with complete mitochondrial genomes available in GenBank to determine the position of *S. peinani*. The construction of maximum-likelihood (ML) tree was completed in phyml 3.0 (Guindon et al. 2010). Ultrafast bootstrap was used to test branch support values with 1000 replicates. They used the complete mt-genomes. The related species are as follows: *Naja atra*, *N. naja*, *N. kaouthia*, *Ophiophagus hannah*, *Sinomicrurus macclellandi*, *Bungarus fasciatus*, *B. multicinctus*, *Emydocephalus ijimae*, *Hydrophis curtus*, *Laticauda semifasciata*, *L. colubrina*, *L. laticaudata*, and *Gloydius blomhoffi.* An ML tree was constructed based on the dataset by online tool RAxML (Kozlov et al. 2019), and the multiple alignment was trimmed with trimAl v1.2 (Capella-Gutierrez et al.^2009^). Results of the phylogenetic analysis show that *S. peinani* is closest to *S. macclellandi*, the similarity is 87.03%, and they are more closely related to cobras, to the exclusion of kraits and other elapids (Figure 1). The newly determined mitogenome of *S. peinani* can enrich analyses of mitochondrial genome variation and the evolutionary relationships of Sinomicrurus and other elapid snakes.

**Figure 1.**
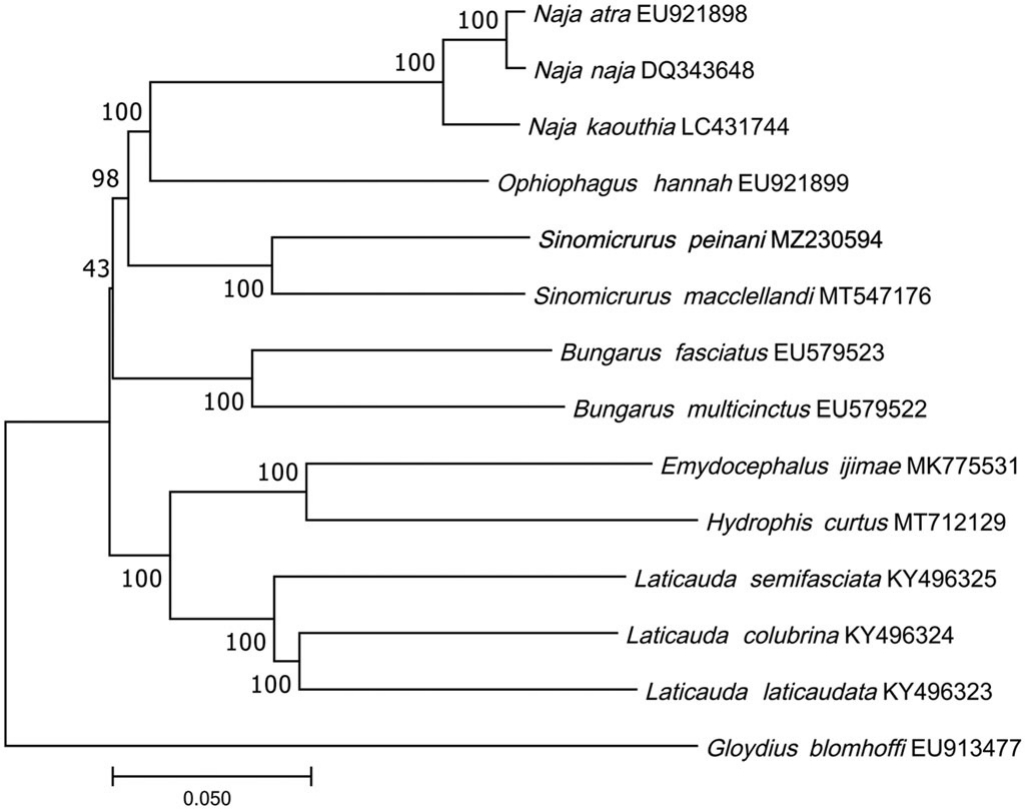
A maximum-likelihood (ML) tree of the *Sinomicrurus peinani* in this study and other 13 related species. Node numbers represent ultra-fast bootstrap percentages, scale bar shows relative branch length.

## Ethical approval

This study has been reviewed and approved by the ‘Experimental animal welfare and ethics review committee of Shenyang Agricultural University’. Reference number: 2021051502.

## Author contributions

Sheng-bo Zhou has completed the collection of experimental samples and main experimental operations, processed the experimental data, and completed the writing and revision of the main part of the paper. Zhao-bo Zhang played an auxiliary role in the experimental operation of this article. Zhi-he Zhang has completed the revision of the layout and assisted in the processing of the test samples. Xin-yu Liu completed the revision of the language in this article. Ping Guan provided the main idea and design, and reviewed the authenticity of the experimental data. Bo Qu helped complete the idea and design of the idea, and conducted the final review of the version to be released. All authors agree to be accountable for all aspects of the work.

## Geolocation information

The location of this study is Shenyang, Liaoning Province, China.

## Data Availability

The data that support the analysis and results of this study are openly available in GenBank with accession number (MZ230594) (http://www.ncbi.nlm.nih.gov/). The associated BioProject, SRA, and Bio-Sample numbers are PRJNA730204, SRR14585256, and SAMN19223701, respectively.
